# Monitoring in emotion regulation: behavioral decisions and neural consequences

**DOI:** 10.1093/scan/nsaa001

**Published:** 2020-03-28

**Authors:** Shirel Dorman Ilan, Roni Shafir, Jeffrey L Birk, George A Bonanno, Gal Sheppes

**Affiliations:** 1 Department of psychology, School of Psychological Sciences, Tel Aviv University, Tel Aviv 6997801, Israel; 2 The Child Psychiatry Division, Edmond and Lily Safra Children’s Hospital, Sheba Medical Center, Tel Hashomer 5266202, Israel; 3 Department of psychology, Teachers College, Columbia University, New York, NY 10027, USA; 4 Department of psychology, Columbia University Irving Medical Center, New York, NY 10027, USA; 5 Sagol School of Neuroscience, Tel Aviv University, Tel Aviv 6997801, Israel

**Keywords:** emotion regulation, distraction, reappraisal, monitoring, consequences, late positive potential

## Abstract

Monitoring and deciding how to adjust an active regulatory strategy in order to maximize adaptive outcomes is an integral element of emotion regulation, yet existing evidence remains scarce. Filling this gap, the present study examined core factors that determine behavioral regulatory monitoring decisions and the neuro-affective consequences of these decisions. Using a novel paradigm, the initial implementation of central downregulation strategies (distraction, reappraisal) and the emotional intensity (high, low) were manipulated, prior to making a behavioral decision to maintain the initial implemented strategy or switch from it. Neuro-affective consequences of these behavioral decisions were evaluated using the Late Positive Potential (LPP), an electro-cortical measure of regulatory success. Confirming predictions, initial implementation of reappraisal in high intensity and distraction in low intensity (Strategy × Intensity combinations that were established in prior studies as non-preferred by individuals), resulted in increased behavioral switching frequency. Neurally, we expected and found that in high (but not low) emotional intensity, where distraction was more effective than reappraisal, maintaining distraction (relative to switching to reappraisal) and switching to distraction (relative to maintaining reappraisal) resulted in larger LPP modulation. These findings suggest that monitoring decisions are consistent with previously established regulatory preferences and are associated with adaptive short-term neural consequences.

## Introduction

Imagine getting stuck in a massive traffic jam while driving to work. Anticipating being late, you feel that anger rises. Wanting to regulate your anger, you reappraise the situation by thinking that traffic jams often look worse than they are. While monitoring how anger regulation is faring, you realize that current reappraisal attempts are not working well. This poses a dilemma of whether to continue reappraising or try a different course of action. You decide to switch to a different option and attempt to distract yourself by listening to the radio. Although still feeling some anger, you notice a sense of relief.

In thinking about the aforementioned situation, several factors can determine monitoring regulatory decisions to maintain a particular strategy or to switch from it to a different one, and their affective consequences. These factors may include elements of the emotional situation, such as the intensity of anger that is activated by the degree of traffic congestion, and the regulatory strategies one is monitoring, such as distraction *vs* reappraisal. Although monitoring appears fundamental in emotion regulation, existing studies remain scarce and indirect with regard to factors that determine monitoring decisions and their affective consequences.

Recent conceptual accounts suggest that emotion regulation is composed of several interacting and iterating valuation systems of key regulatory stages, in which emotion regulation-related decisions are made (e.g. Bonanno and Burton, 2013; Gross, 2015; Sheppes, 2020). Identification involves making the initial decision whether to regulate an emotion or not. If a decision to regulate was reached, a selection regulatory stage involves choosing one of several available regulatory strategies. Following the selection of a particular strategy, it is executed during an implementation stage. Only then monitoring begins, involving the decision whether and how to adjust an active implemented strategy across subsequent iterations, in order to maximize adaptive outcomes.

The conceptual extended process model of emotion regulation (Gross, 2015) specifies three monitoring decision options: (i) Maintenance: a decision to continue implementing a currently active strategy, conceptually involves subsequent iterations where regulation is positively valued during identification, and the specific active strategy is positively valued during selection. (ii) Switching: a decision to alter a currently active implemented strategy, conceptually involves subsequent iterations where regulation is positively valued during identification, but an alternative regulatory strategy is positively valued during selection. (iii) Stopping: a decision to cease regulation altogether, conceptually involves a subsequent iteration where regulation is negatively valued during identification. This model further argues that adequate monitoring decisions lead to adaptive outcomes.

Moving from conceptual grounds, existing empirical monitoring findings remain indirect. Paret *et al.* (2011) empirically supported a conceptual model (Kalisch, 2009), by providing neural correlates of regulatory maintenance across time. While important, these studies do not describe the switching and stopping monitoring decision options or how individuals decide between options.

Extending the scope beyond maintenance, one type of indirect studies (e.g. Yoon and Joormann, 2012; Peuters *et al.*, 2019) examined the affective consequences of forced switching (i.e. instructing participants to implement two different strategies consecutively) and maintenance (i.e. instructing participants to implement the same strategy twice). While important, because participants could not freely decide between maintaining and switching, factors that influence monitoring decisions remain unexplored.

A second type of correlational studies indirectly examined individuals’ decisions to maintain or switch from an initial implemented strategy. Kato (2012) found that individuals who reported more switching from an inefficient implemented strategy and reported decreased levels of psychopathology. Recently, utilizing experience sampling, Kalokerinos *et al.* (2017) demonstrated that switching from regulatory strategies that were inefficiently reduced negative affect, subsequently led to improved affect. Although important, these correlational studies that did not manipulate factors influencing monitoring decisions cannot reach causal conclusions.

Conceptual advances suggest potential factors that influence monitoring decisions (Bonanno and Burton, 2013). This framework focuses on individual differences in the sensitivity to the internal (e.g. subjective and physiological emotional states) and external (e.g. contextual or social cues) environment, which may influence the decision between the three core monitoring decision options (maintain, switch, stop).

Empirical support for this framework comes from a single study that examined the influence of the internal environment on the decision to maintain *vs* switch from an implemented strategy and its consequences for general well-being (Birk and Bonanno, 2016). Specifically, participants were asked to implement distraction or reappraisal while being physiologically monitored and were then given a choice between maintenance and switching.

Results showed that increased internal physiological intensity while implementing reappraisal, which denotes inefficient regulation, was associated with increased switching to distraction. Furthermore, participants who showed high correspondence between increased internal physiological intensity during reappraisal implementation and switching from reappraisal reported higher well-being.

While valuable, Birk and Bonanno (2016) did not manipulate participants’ internal intensity and therefore could not evaluate the causal influence of this fundamental factor on monitoring decisions. Furthermore, focusing on the role of the internal environment leaves the important role of the external environment unexplored. Last, the evaluation of adaptive consequences of monitoring decisions was based on participants’ self-reported well-being rather than on immediate behavioral or neural affective consequences. Despite many advantages, self-reports represent the endpoint, rather than online underlying mechanism, of emotional modulation and are susceptible to reporting biases.

Overcoming these limitations, the present study provided two important contributions to the scant literature on regulatory monitoring. The first goal was to provide causal (rather than correlational) evidence for the influence of two core interconnected factors on monitoring regulatory decisions. The second goal was to provide evidence for neuro-affective (rather than self-reported) consequences of monitoring decisions.

We utilized our conceptual framework (Sheppes, 2020) that focuses on the combination of: (i) external generated emotional intensity (high, low) and (ii) regulatory strategy (distraction, reappraisal). Our conceptual framework has successfully explained the role of these two core factors in two other regulatory stages that precede the post-implementation/monitoring stage, namely, implementation and selection stages.

In the implementation regulatory stage, our framework (Sheppes and Gross, 2011) and supporting findings (e.g. Sheppes and Meiran, 2007; Shafir *et al.,* 2015) indicated that in high-intensity situations, early attentional disengagement via distraction leads to a stronger emotional modulation, relative to reappraisal, which involves engaging with emotional information prior to a late semantic meaning reinterpretation. By contrast, in low-intensity situations, distraction and reappraisal are equally effective, but only reappraisal, that involves making sense of emotional events, may provide long-term benefits (Wilson and Gilbert, 2008; Thiruchselvam *et al.,* 2011).

In the selection regulatory stage, our model (Sheppes and Levin, 2013) and supporting evidence (e.g. Sheppes *et al.,* 2014; Shafir *et al.,* 2016) repeatedly found that in high intensity, most individuals prefer distraction, which results in enhanced short-term emotional modulation, over reappraisal, and in low-intensity, most individuals prefer reappraisal, which may provide long-term benefits, over distraction. These regulatory preferences appear very robust (Cohen’s *d* = ~2, with 90% of individuals showing these patterns, see Sheppes, 2020 for review).

Drawing from these lines of research, our first research question was whether external emotional intensity (high, low) and regulatory strategies (distraction, reappraisal) influence regulatory decisions in a monitoring stage. To that end, we created a novel experimental paradigm that manipulates these two independent variables. In each trial, participants were initially instructed to implement distraction or reappraisal when facing images of low or high intensity (henceforth ‘initial implementation’) and were then asked to choose whether they wish to maintain the initial implemented strategy or switch from it (henceforth ‘monitoring choice’). Participants then implemented their chosen option (henceforth ‘post-choice implementation’).

Consistent with our conceptual framework and previous findings (Sheppes, 2020), our first hypothesis was that initial implementation that is incongruent with averaged regulatory preferences obtained in prior studies (i.e. reappraisal in high intensity, distraction in low intensity) would result in increased switching frequency, relative to initial implementation that is congruent with previously established averaged regulatory preferences (i.e. distraction in high intensity, reappraisal in low intensity).

Our second research question was what are the neuro-affective consequences of monitoring decisions. To answer it, we utilized event-related potentials (ERPs), which have been extensively used in the study of emotion regulation (e.g. Hajcak *et al.,* 2012). We focused on the late positive potential (LPP), a centro-parietal electro-cortical component that reflects enhanced processing of emotionally arousing information. Attenuation of this component reflects downregulation success (Dennis and Hajcak, 2009) with good internal consistency (Moran *et al.*, 2013).

We first wished to replicate prior neural findings (Shafir *et al.,* 2015) by demonstrating that in high (but not low) intensity, initial distraction implementation would result in greater LPP modulation, relative to reappraisal. Importantly, this study was the first to examine the neuro-affective consequences (LPP modulation) of monitoring regulatory decisions. We hypothesized that exclusively in high intensity, where neural differences between distraction and reappraisal are evident (Shafir *et al.,* 2015), maintaining distraction (relative to switching to reappraisal) and switching to distraction (relative to maintaining reappraisal) would each be associated with stronger LPP modulation.

## Method

Below we report how we determined our sample size, all data exclusions, manipulations and measures in the study.

### Ethical approval

This study was approved by the institutional review board of Tel Aviv University, and participants provided informed consent prior to inclusion in the study.

### Participants

Thirty native Hebrew-speaking^1^ subjects participated. Sample size was pre-determined based on a priori rule of collecting data from 30 participants for ERP studies conducted in our lab (e.g. Shafir *et al.,* 2015; Shafir and Sheppes, 2018; Shafir *et al.*, 2018). Two participants were excluded prior to data analyses. One participant was not a Hebrew native speaker, and another participant did not comply with experimental instructions (see below). The main results reported below remain unchanged when including these two participants (all *P*s < 0.02). The final sample consisted of 28 participants (8 men, mean age = 23.27 years, s.d. = 2.08).

### Stimuli

One hundred eighty negative pictures were chosen from previously validated pictorial datasets^2^ (IAPS: Lang *et al.,* 2008; EmoPicS: Wessa *et al.,* 2010). High-intensity pictures (*n* = 90, *M*_arousal_ = 6.45, *M*_valence_ = 2.04) significantly differed in valence and arousal normative ratings from low-intensity pictures (*n* = 90, *M*_arousal_ = 4.73, *M*_valence_ = 3.38) (both *F*s > 423, *P*s < 0.001; c.f. Sheppes *et al.,* 2011). Picture contents were matched for high- and low-intensity categories when possible. Importantly, analyses that decomposed interactions involving emotional intensity compared different regulation instructions within each intensity separately. Therefore, possible content differences between intensities have no bearing on the results.

### Procedure

Following initial EEG setup, participants learned (four trials) and practiced (eight trials) distraction and reappraisal implementation (c.f., Sheppes *et al.,* 2014). Adherence to regulatory instructions involved asking participants to talk out loud throughout implementation. Distraction instructions involved disengaging attention from emotional pictures by producing unrelated neutral thoughts (e.g. visualizing geometric shapes or daily chores). Reappraisal instructions involved engaging with the processing of emotional pictures, but reinterpreting their negative meaning (e.g. by thinking about less negative aspects of situations or that situations will improve over time) (c.f. Sheppes *et al.,* 2014). Participants were asked not to form reality challenge reappraisals (i.e. interpret emotional events as fake; Qi *et al.,* 2017).

The task consisted of 180 trials (divided into six equally long blocks, separated by breaks). Pictures of low and high intensity were presented in a random order, with no more than two consecutive trials of the same intensity, and were randomly assigned to reappraisal or distraction.

To ensure adherence to regulatory instructions, participants provided five oral examples of each strategy during experimental breaks. Based on a priori exclusion criteria in our lab, participants who made more than 50% errors (*n* = 1) were excluded from data analyses. Average percentage of errors was minimal (*M* = 0.05%, s.d. = 0.08).

**Fig. 1:**
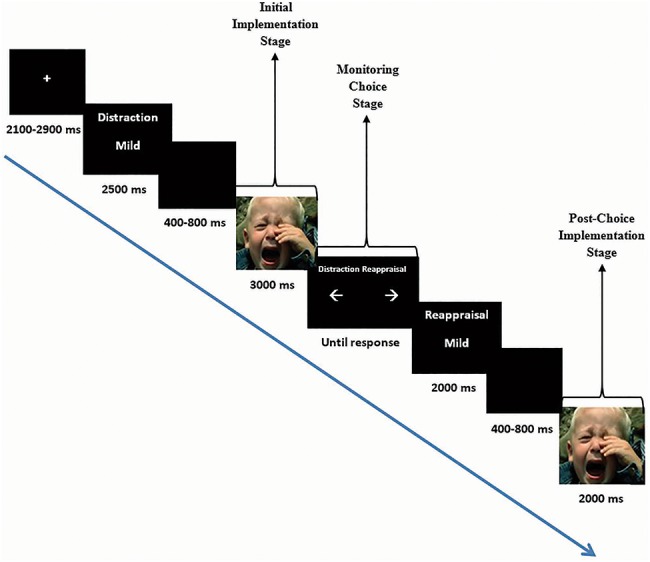
Trial structure: An example of a low-intensity trial, where distraction was the initial implemented regulatory strategy, and the participant chose to switch to reappraisal. Note that this choice pattern is consistent with regulatory preferences for reappraisal over distraction in low intensity.

Each trial (see Figure 1) began with a fixation cross (jittered between 2100 and 2900 ms) followed by a 2500 ms cue screen that signaled the intensity of the upcoming picture (‘Intense’ or ‘Mild’) and the initial strategy implementation (‘Distraction’ or ‘Reappraisal’) (c.f. Shafir *et al.,* 2015), followed by a jittered 400–800 ms black screen. The picture was then presented (3000 ms), during which participants implemented the required strategy (*‘*initial implementation’). Then, a choice screen was presented, where participants were asked to choose whether they wished to maintain the initial implemented strategy (i.e. choosing distraction following initial distraction implementation or choosing reappraisal following initial reappraisal implementation) or switch to the other regulatory option (i.e. choosing distraction following initial reappraisal implementation or choosing reappraisal following initial distraction implementation) (*‘*monitoring choice*’*). Then, a 2000 ms cue screen presented both the chosen regulatory strategy and the intensity of the picture that was previously shown, followed by a jittered 400–800 ms black screen. The same picture was then presented again for 2000 ms, during which participants implemented their chosen strategy *(‘*post-choice implementation’). The post-implementation window was 1000 ms shorter than the initial implementation window to balance adequate duration to observe LPP effects with maintaining a 5-s picture presentation per trial (c.f., Li *et al.,* 2017).

To remind participants that monitoring decisions were aimed at reducing negative experience, the offset of 10% of pictures was followed by a Likert rating scale in which participants reported their negative experience (1 = ‘not negative at all’, 9 = ‘extremely negative’. For complete explanation and analysis of partial self-report data, see Supplementary Materials, page 1.

### Electrophysiological recordings and data reduction

EEG recordings used a Biosemi ActiveTwo recording system (Biosemi B. V., Amsterdam, The Netherlands), from 32 electrodes sites,^3^ and one electrode on each of the left and right mastoids. The horizontal electrooculogram (EOG) was recorded from two electrodes placed 1 cm to the left and right of the external canthi, and vertical EOG was recorded from an electrode placed beneath the left eye. The voltage from each electrode site was referenced online with respect to Common Mode Sense/Driven Right Leg electrodes. EEG data were sampled at 256 Hz.

Offline signal processing entailed EEGLAB and ERPLAB Toolboxes (Delorme and Makeig, 2004; Lopez-Calderon and Luck, 2014). Data from all electrodes were re-referenced to the average activity of the left and right mastoids. Continuous EEG data were then band-pass filtered (cutoffs: 0.05–20 Hz; 12 dB/oct rolloff). Eye movement artifacts were removed using independent component analysis (Delorme and Makeig, 2004; Mennes *et al.,* 2010).

For the initial implementation LPP analysis, EEG was epoched into 3200 ms segments, starting 200 ms (baseline) before the picture appeared on the screen and lasting 3000 ms (end of the initial implementation). Similarly, for the post-choice implementation LPP analysis, EEG was epoched into 2200 ms segments, starting 200 ms (baseline) before the picture re-appeared on the screen and lasting 2000 ms (end of post-choice implementation). All trials containing activity exceeding 80 μV within 200 ms were excluded.

The initial implementation LPP was defined as the mean amplitude between 300 (when the LPP becomes evident; Hajcak *et al.,* 2010) and 3000 ms (end of the initial implementation stage). Similarly, the post-choice implementation LPP was defined as the mean amplitude between 300 and 2000 ms (end of the post-choice implementation stage). The LPP was measured as the average activity over centro-parietal electrode sites, where the LPP is typically maximal (CPz–CP1–CP2; c.f., Paul *et al.,* 2015; Thiruchselvam *et al.,* 2011).

### Statistical analyses

Preliminary initial implementation analyses include data that precede monitoring decisions, where all analyzed factors are experimentally manipulated, resulting in the total number of trials (*n* = 180) equally divided across four conditions (*n* = 45 per condition). Slight variation in trial numbers across conditions is possible due to differential ERP trial rejection. However, rejections were minimal [Valid trials: low intensity/distraction: *M* = 43.96, s.d. = 2.07; low intensity/reappraisal: *M* = 44.21, s.d. = 1.52; high intensity/distraction: *M* = 44.10, *s.d.* = 1.73; high intensity/reappraisal: *M* = 44.18 trials, s.d. = 1.48]. Trial number did not significantly differ between conditions (all *F*s < 1). Accordingly, to replicate prior implementation findings, preliminary initial implementation analyses employed a 2 × 2 analysis of variance (ANOVA) with emotional intensity (high, low) and initial implementation (distraction, reappraisal) as repeated-measures factors and LPP as a dependent variable.^4^

Trial numbers across conditions that constitute the first research question (behavioral regulatory monitoring decisions) are matched by experimental design. Accordingly, we employed a 2 × 2 ANOVA with emotional intensity (high, low) and initial implementation (distraction, reappraisal) as repeated-measures factors and switching frequency as a dependent variable.

To examine the second research question (short-term neural consequences of monitoring decisions), we first created a neural consequence LPP outcome variable. For each trial, we subtracted the post-choice implementation LPP amplitude from the respective initial implementation/pre-choice LPP amplitude in that trial, with higher scores indicating stronger LPP attenuation (i.e. higher regulatory success). Note that each trial consists of a pre-choice implementation phase and a post-choice implementation phase and thus the subtraction that constitutes the dependent variable occurs within each individual trial. The neural consequence LPP variable was created to adjust for initial implementation LPP differences between distraction and reappraisal (see results below and c.f. Shafir *et al.,* 2016). Notably, the main results reported below remain unchanged when re-conducting the analysis on the post-choice implementation LPP, without performing these subtractions (i.e. the predicted Emotional Intensity × Initial Implementation × Monitoring Choice interaction remains significant [*b* = 3.82, SE = 1.72, 95% CI (0.45, 7.20), *F*(1, 4254) = 4.94, *P* = 0.026]).

Because the neural consequence measure takes into account LPPs that are measured following monitoring decisions, trial numbers across conditions cannot be experimentally controlled (Table 1 for all values). This element potentially biases conventional ANOVAs. Accordingly, the analyses of the second research question were performed on individual trials (rather than on condition averages across trials) of the neural consequence LPP, using linear mixed models (LMMs, using PROC MIXED procedure in SAS version 9.4 for Windows; Galwey, 2014; Boisgontier and Cheval, 2016). LMM is a widely accepted method that accounts for unequal trial numbers in experimental designs, by treating between-subject variance in the outcome measure as a random effect, in addition to modeling the within-subjects effects (e.g. Cohen *et al.*, 2011; Radel *et al.*, 2018; Smedley and Smith, 2018). LMMs also make use of all available data, which protects from reduced power and reliability of averaged estimates of cells with considerable amount of discarded observations.

**Table 1:** Trial means, standard deviations, max and min values for each experimental condition in the post-choice implementation analyses

	High Intensity				Low Intensity			
	Dist→Dist	Reap →Dist	Dist→Reap	Reap→Reap	Dist→Dist	Reap→Dist	Dist→Reap	Reap→Reap
Average	34.57	20.43	10.43	24.54	27.07	8.50	17.89	36.46
SD	7.12	8.95	7.12	8.92	9.53	6.07	9.49	6.10
Max	45	42	25	41	44	29	36	44
Min	20	4	0	3	9	1	1	16

Note: Repeated-measures factors include emotional intensity (high, low), initial regulatory strategy (distraction, reappraisal) and monitoring regulatory choice (maintain, switch), and the dependent variable is ‘neural consequence’ LPP.

Note: Dist→Dist: maintaining distraction following initial distraction implementation; Reap→Dist: switching to distraction following initial reappraisal implementation; Dist→Reap: switching to reappraisal following initial distraction implementation; Reap→Reap: maintaining reappraisal following initial reappraisal implementation.

Note: The LPP is considered a large robust component, and even in our smallest cell the average exceeds the recommended number of trials required to produce a reliable LPP (Moran *et al.,* 2013).

Our LMM modeling approach involved balancing accuracy with parsimony by starting with a maximum random effect structure, followed by separate steps that decrease in complexity (first examining the random three-way interaction, then random two-way interactions, then random main effects), involving the removal of random effects not supported by the data. Each step applies multiple iterations and likelihood evaluations to achieve convergence of the final model estimates. At the end of this convergence process, there are cases when the final model estimates a random effect as one of its boundary constraints, such as exactly zero. We adopted a conservative approach of only removing random effects that explained exactly zero variance c.f., Bates *et al.,* 2015 (see Supplementary Table S3 for all non-zero random effects). Kenward–Roger approximation for degrees of freedom, which entails a Satterthwaite approximation (SAS Institute Inc., 2013), was computed as recommended for unbalanced designs (Schaalje *et al.*, 2001). We removed correlations between random effects to achieve model convergence.

The initial maximum random effect structure converged (−2*LL = 42705.9, *AIC* = 42731.9). In the first model complexity reduction step, the random effect of the Emotional Intensity × Initial Implementation × Monitoring Choice three-way interaction was estimated to be zero. Therefore, in the next model complexity reduction step, this random interaction was removed. This new model converged with unchanged fit statistics (−2*LL = 42705.9, *AIC* = 42731.9) while also revealing that the two-way interaction of Emotional Intensity × Initial Implementation was zero. Therefore, in the next model complexity reduction step, this random interaction was removed. This new model converged with unchanged fit statistics as before (−2*LL = 42705.9, *AIC* = 42731.9) and revealed that the main effect of monitoring choice was zero.

Accordingly, the final model consisted of all fixed effects together with the following random effect structure: intercept, Emotional Intensity, Initial Implementation, Initial Implementation × Monitoring Choice and Emotional Intensity × Monitoring Choice. This final model converged (−2*LL = 42705.9, *AIC* = 42731.9) and had comparable model fit to a similar model that allowed random effects to correlate (−2 log likelihood = 42693.5, AIC = 42735.5; *Δ*−2*LL = 12.4, *df* = 10, *P* = 0.26).

## Results

### Replicating prior neural findings during initial implementation

**Fig. 2:**
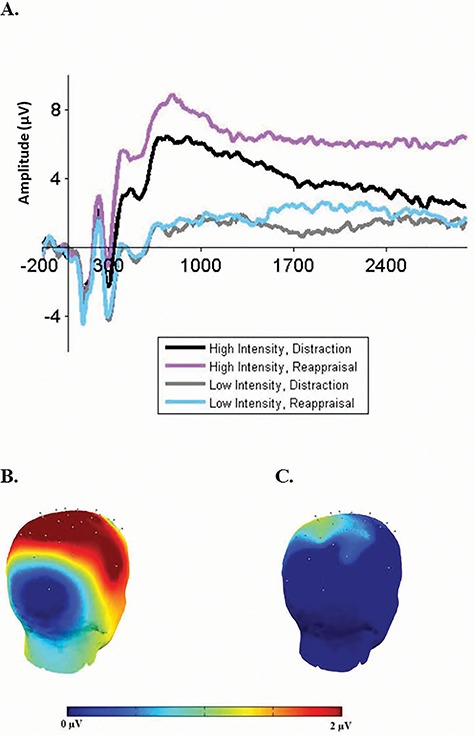
(A) Initial implementation findings. LPP amplitudes during initial implementation of distraction and reappraisal in high and low emotional intensities. Waveforms are averages across CPz, CP1 and CP2 electrodes. The *x*-axis runs from the beginning of the baseline (200 ms before picture onset) to the end of the picture presentation (3000 ms). (B, C) Head maps of the LPP topographical distribution. Voltage difference score for the initial implementation of distraction and reappraisal in high (B) and low (C) emotional intensities was calculated as: (averaged initial reappraisal implementation)−(averaged initial distraction implementation).

We first wished to replicate prior findings (e.g. Shafir *et al.,* 2015) demonstrating that in high (but not low) emotional intensity, initial implementation of distraction would result in greater LPP modulation, relative to reappraisal. The ANOVA yielded a predicted Emotional Intensity × Initial Implementation interaction that was marginally significant [*F*(1, 27) = 3.36, *P* = 0.07, η_p_^2^ = 0.11; Figure 2, Supplementary Table S1 for all effects]. Follow-up analyses supported predictions in showing that in high intensity [*F*(1, 27) = 13.43, *p* = 0.001, η_p_^2^ = 0.33], distraction implementation resulted in decreased LPPs (*M* = 4.07, SE = 1.08), relative to reappraisal implementation (*M* = 6.50, SE = 0.97). As expected, in low intensity, there were no differences [*F*(1, 27) < 1, *P =* 0.45, η_p_^2^ = 0.02] in LPPs between distraction (*M* = 0.93, SE = 0.92) and reappraisal implementation (*M* = 1.58, SE = 0.86).

### Regulatory preferences predict regulatory choices to switch *vs* maintain an implemented strategy during post-implementation monitoring

Our first research question examined whether initial implementation that is incongruent with regulatory preferences (i.e. distraction in low intensity, reappraisal in high intensity) results in increased switching frequency, relative to initial implementation that is congruent with regulatory preferences (i.e. reappraisal in low intensity, distraction in high intensity)?

**Fig. 3:**
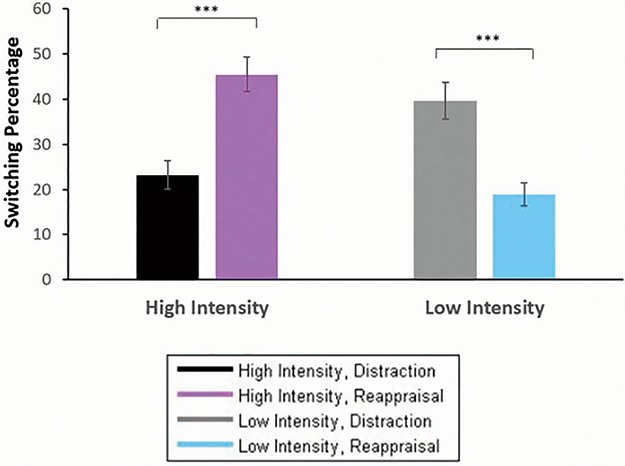
Percentage of trials (*y*-axis) during which participants chose to switch (*vs* maintain) following initial implementation of distraction and reappraisal in high and low emotional intensities. ^***^P < 0.001.

Prior to hypothesis testing, we confirmed using ANOVAs previously established regulatory preferences, in finding that in a monitoring context, intensity increase from low-to-high was associated with increased preference for distraction over reappraisal (i.e. increased preference to maintain distraction or switch to distraction from reappraisal) [*t*(27) = −7.81, *P* < 0.001, *d =* 1.47]. Confirming our main prediction, we found a significant Emotional Intensity × Initial Implementation interaction [*F*(1, 27) = 60.99, *P* < 0.001, η_p_^2^ = 0.69; see Figure 3 and Supplementary Table S2 for all effects]. Follow-up analyses showed that in high intensity, initial reappraisal implementation (the non-preferred strategy) resulted in higher switching frequency (*M* = 45.41%, SE = 0.04), compared to initial distraction implementation (the preferred strategy, *M* = 23.22%, SE = 0.03) [*F*(1, 27) = 16.14, *P* < 0.001, η_p_^2^ = 0.37]. A mirrored pattern emerged in low intensity, where initial distraction implementation (the non-preferred strategy) resulted in higher switching frequency (*M* = 39.62%, SE = 0.40), compared to initial reappraisal implementation (the preferred strategy, *M* = 18.87%, SE = 0.02) [*F*(1, 27) = 19.68, *P* < 0.001, η_p_^2^ = .42].

### Neuro-affective consequences of monitoring regulatory decisions

Our second research question examined the short-term neural consequences (LPP modulation) of monitoring regulatory choices. We expected to show that in high intensity, where distraction is more effective than reappraisal (Sheppes and Meiran, 2007; Shafir *et al.,* 2015, 2016), choosing to maintain (or switch to) distraction would result in greater LPP modulation, compared with choosing to maintain (or switch to) reappraisal. By contrast, in low intensity, where distraction and reappraisal are equally effective, choosing one strategy over the other was not expected to result in differential LPPs.

**Fig. 4:**
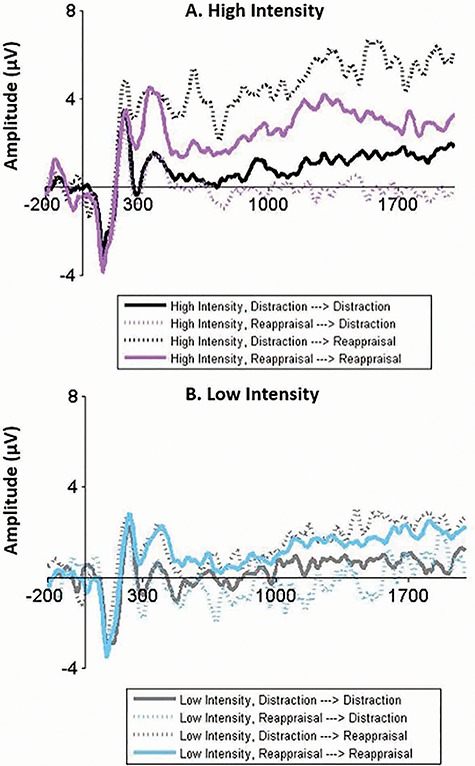
(a,b) Post-choice implementation findings. LPP amplitudes during post-choice implementation for distraction and reappraisal in high (A) and low (B) emotional intensities. The *x*-axis runs from the beginning of baseline (200 ms before picture onset) to the end of the picture presentation (2000 ms). Note that these waveforms represent post-choice implementation LPP averaged amplitudes without subtractions from initial implementation LPP amplitudes, which were only performed for the statistical analyses. Notably, higher LPP amplitudes represent decreased emotional modulation (i.e. lower regulatory success). Note: Distraction→Distraction: maintaining distraction following initial distraction implementation; Reappraisal→Distraction: switching to distraction following initial reappraisal implementation; Distraction→Reappraisal: switching to reappraisal following initial distraction implementation; Reappraisal→Reappraisal: maintaining reappraisal following initial reappraisal implementation. (c) Post-choice implementation findings. Head maps of the LPP topographical distribution during post-choice implementation of distraction and reappraisal for high (A, B) and low (C, D) emotional intensities. (A) and (C) were calculated as: (averaged initial distraction implementation followed by post-choice reappraisal implementation)−(averaged initial distraction implementation followed by post-choice distraction implementation). (B) and (D) were calculated as: (averaged initial reappraisal implementation followed by post-choice reappraisal implementation)−(averaged initial reappraisal implementation followed by post-choice distraction implementation). Note: Distraction→Distraction: maintaining distraction following initial distraction implementation; Reappraisal→Distraction: switching to distraction following initial reappraisal implementation; Distraction→Reappraisal: switching to reappraisal following initial distraction implementation; Reappraisal→Reappraisal: maintaining reappraisal following initial reappraisal implementation.

Results using LMMs showed the expected Emotional Intensity × Initial Implementation × Monitoring Choice interaction [*b* = 5.99, SE = 2.50, 95% CI (1.09, 10.89), *F*(1, 3653) = 5.75, *P* = 0.017, Figure 4 for LPP waveforms and LPP topographical distribution and Supplementary Table S3 for all effects].^5^ Follow-up analyses explored lower-order effects in the context of a full model (using ‘estimate’ statements in the SAS syntax). Despite having clear a priori predictions, when decomposing this three-way interaction, we corrected for multiple comparisons by applying the well-established Benjamini–Hochberg procedure that adjusts the criterion for significance by controlling for the false discovery rate (Benjamini and Hochberg, 1995).

Decomposing the three-way interaction revealed that, consistent with our prediction, in high intensity [*b* = 8.22, SE = 1.84, 95% CI (4.60, 11.83), *t*(246) = 4.48, *P* < 0.001, α_adjusted_ = 0.013], but not in low intensity [*b* = 2.23, SE = 1.88, 95% CI (−1.47, 5.92), *t*(306) = 1.19, *P* = 0.237, α_adjusted_ = 0.050], there was an Initial Implementation × Monitoring Choice interaction, such that choosing to maintain (or switch to) distraction, relative to maintain (or switch to) reappraisal, resulted in larger LPP modulation. Specifically, in high intensity, switching to distraction following initial reappraisal implementation resulted in substantially stronger LPP modulation (*M* = 7.03, SE = 1.10), relative to maintaining reappraisal (*M* = 2.62, SE = 0.94) [*b* = 4.41, SE = 1.20, 95% CI (2.02, 6.80), *t*(93.3) = 3.66, *P* < 0.001, α_adjusted_ = 0.025]. Complimentary, in high intensity, maintaining distraction following initial distraction implementation resulted in stronger LPP modulation (*M* = 2.95, SE = 0.86), relative to switching to reappraisal (*M* = −0.85, *SE* = 1.49) [*b* = 3.81, SE = 1.51, 95% CI (0.80, 6.81), *t*(74.9) = 2.52, *P* = 0.014, α_adjusted_ = 0.038]. A similar pattern of findings emerged when using an alternative LMM with a more minimal random effect structure or a repeated-measures ANOVA (Supplementary Material, pages 4–7).

## Discussion

While a monitoring regulatory stage is considered an integral part of emotion regulation and successful functioning, empirical evidence remains scarce. Our first research question was whether previously established regulatory preferences (i.e. the combination of emotional intensity and initial regulatory strategy) influence the decision to maintain *vs* switch from the implemented strategy. Supporting predictions, initial implementation that is incongruent with regulatory preferences (i.e. distraction in low intensity, reappraisal in high intensity) resulted in increased switching frequency, relative to initial implementation that is congruent with regulatory preferences (i.e. reappraisal in low intensity, distraction in high intensity). Our second research question was what are the neuro-affective consequences (LPP modulation) of monitoring regulatory decisions. We predicted and found that in high (but not low) emotional intensity, where distraction is more effective than reappraisal, choosing distraction (either by maintaining or switching to distraction) resulted in adaptive neural consequences (i.e. greater LPP modulation).

Considering our first research question, results extend our conceptual account and selection findings (Sheppes, 2020), by elucidating the role of regulatory preferences for the unexplored monitoring regulatory stage. In each emotional intensity, initial implementation that is incongruent with regulatory preferences, led to increased switching, relative to initial implementation that is congruent with regulatory preferences.

Although monitoring decisions were strongly determined by regulatory preferences, we also observed considerable inertial effects. Even in cases where the initial implemented strategy was non-preferred and less effective (reappraisal in high intensity), it was nonetheless maintained in a notable amount of trials. It seems that an initial implemented strategy may function as a strong default. This notion is further supported by studies demonstrating that presenting individuals with a ‘default’ option leads to disproportionately sticking with it (e.g. Hartman *et al.*, 1991; Suri *et al.*, 2015).

Considering our second research question, we evaluated adaptive consequences of monitoring decisions using neural measures, transcending prior self-report findings (Birk and Bonanno, 2016). These results extend prior neural findings (Shafir *et al.,* 2015) by showing that in the monitoring regulatory stage, choosing to maintain (or switch to) distraction in high intensity results in adaptive neural consequences. These results are consistent with the notion that in high intensity, early attentional disengagement via distraction is more effective in the short term, compared to late operating reappraisal (Sheppes and Gross, 2011).

Accordingly, distraction may serve as a ‘first aid’ tool in highly intense situations, not only during initial implementation (Shafir *et al.,* 2015) but also following monitoring decisions. Notably, however, distraction has significant shortcomings in the long term (Wilson and Gilbert, 2008). Thus, future studies should examine the benefits of maintaining reappraisal in the long term, where reappraisal is predicted to be more beneficial (e.g. Thiruchselvam *et al.,* 2011).

Our results have clinical implications. Repeated failure to make flexible monitoring decisions that are sensitive to contextual demands (i.e. failing to switch from inefficient strategies or failing to maintain efficient strategies) constitutes a form of emotional dysregulation that may be associated with psychopathology (Sheppes *et al.,* 2015). However, studies investigating monitoring decisions and their consequences in clinical populations are crucially needed.

The current study has several limitations. First, we focused on the combination of two elements—external negative emotional intensity (high, low) and two regulatory strategies (distraction, reappraisal). Based on our conceptual framework (Sheppes and Levin, 2013) and prior findings (Shafir *et al.,* 2015), we had the clearest predictions regarding these two elements. Nonetheless, future studies should explore additional factors, including positive emotional intensity and other strategies that may influence monitoring decisions.

Second, we focused on two monitoring decision options (maintaining and switching), leaving the third option—stopping to regulate—unexplored. Future studies should examine factors that determine decisions to stop regulating and the neuro-affective consequences of stopping.

Third, our a priori design decisions led to being able to evaluate neural, but not subjective experience consequences, of regulatory decisions. To evaluate consequences of regulatory decisions, one has to obtain pre-choice and post-choice implementation indices (c.f., Shafir *et al.,* 2016). The pre-choice index is crucial to account for well-established differences between reappraisal and distraction during initial implementation (e.g. Shafir *et al.,* 2015; see also a replication above). While our design involved pre-choice and post-choice LPP measurements (collected continuously and unobtrusively), we did not collect a pre-choice measure of self-reported negative experience. This a priori decision (c.f., all prior regulatory selection studies, e.g. Sheppes *et al.,* 2011, 2014) to refrain from asking participants to provide pre-choice ratings immediately prior to making regulatory decisions is based on a concern that this explicit reporting will bias naturally occurring choices. Conceptually, providing baseline self-reports shifts the focus from examining our causal externally manipulated intensity to the examination of the influence of (measured) internal intensity (Birk and Bonanno, 2016).

Fourth, choosing to maintain (or switch to) reappraisal under low intensity may not necessarily reflect a clear preference for reappraisal. Participants may prefer distraction-over-reappraisal under high intensity, because distraction is more effective than reappraisal. However, given that in low intensity, there are no short-term efficacy differences between the strategies, participants may prefer reappraisal because they strive to balance their overall preferences, or they feel they are expected to use both strategies. More generally, providing participants with only two decision options yields choice preferences that are not fully independent from one another.

Last, prior to initial implementation, participants received information regarding the emotional intensity and the instructed strategy. This may have influenced participants’ later monitoring decisions. However, consistent with prior paradigms (Shafir *et al.,* 2015, 2018), providing information on both variables equates the saliency of each prior to upcoming monitoring decision.

## Funding

G. Sheppes is supported by the Israel Science Foundation (Grant No. 1130/16).

## Conflict of interest

None declared.

## Supplementary Material

scan-18-207-File008_nsaa001

## Footnotes

1 We set an a priori native Hebrew proficiency inclusion rule, because understanding and implementing complex cognitive emotion regulation strategies require high verbal proficiency (c.f., Sheppes *et al.,* 2011, 2014).

2 Picture codes were as follows: Low intensity: IAPS: 224, 1111, 1270, 1271, 1274, 1275, 1280, 1301, 2120, 2278, 2312, 2399, 2456, 2457, 2490, 2590, 2682, 2691, 2692, 2700, 2718, 2722, 2753, 2795, 3216, 3280, 6010, 6190, 6561, 6562, 6825, 6836, 7092, 7135, 7359, 7360, 7361, 7520, 7521, 8231, 9001, 9002, 9008, 9010, 9031, 9041, 9045, 9046, 9090, 9101, 9102, 9110, 9120, 9145, 9160, 9171, 9180, 9182, 9168, 9190, 9270, 9280, 9290, 9291, 9330, 9331, 9341, 9342, 9360, 9373, 9390, 9404, 9415, 9417, 9726, 9427, 9440, 9445, 9452, 9469, 9470, 9471, 9530, 9584, 9590, 9592, 9594, 9610, 9912, 9926. High intensity: EmoPicS: 209, 210, 212, 231, 232, 233, 234, 235, 236, 238, 239, 240, 241, 242, 243, 244, 245, 246, 247, 248, 249, 250, 251, 252; IAPS: 2053, 2730, 2800, 3000, 3005.1, 3010, 3015, 3030, 3060, 3064, 3068, 3071, 3080, 3100, 3101, 3102, 3110, 3120, 3130, 3140, 3150, 3168, 3170, 3180, 3230, 3261, 3266, 3350, 3400, 3530, 3550, 6212, 6230, 6312, 6313, 6315, 6350, 6360, 6510, 6520, 6540, 6550, 6570, 6831, 6838, 9040, 9050, 9181, 9183, 9250, 9252, 9300, 9400, 9410, 9412, 9413, 9420, 9500, 9570, 9571, 9600, 9635.1, 9908, 9910, 9911, 9921.

3 The 32 EEG scalp electrodes sites were as follows: Fp1, Fp2, Fpz, Af3, Af4, Afz, F1, F3, Fz, Fc1, Fc2, Fcz, C1, C3, C2, C4, Cz, Cp1, Cp2, Cpz, P1, P3, P2, P4, Pz, O1, O2, Poz, T7, T8, F2, F4.

4 A significance level of 5% (two-sided) was selected for the two analyses of variance (ANOVAs) used to replicate prior implementation findings and to examine the first research question (behavioral regulatory monitoring decisions). In both ANOVAs, we used only 2 × 2 (or 2 × 2 × 2) experimental designs, which do not require any tests of sphericity (Mordkoff, 2015).

5 To mitigate concerns that our findings depend on a large DF value, we also tested the same model using a between-within (i.e. ddfm = BETWITHIN) minimal value of degrees of freedom. Results in this alternative model remained identical, including the three-way interaction of interest [*b* = 5.99, SE = 2.48, 95% CI (0.88, 11.10), *F*(1, 25) = 5.83, *P* = 0.023].
