# Supplementary material for: Monitoring in emotion regulation: behavioral decisions and neural consequences
Source: Soc Cogn Affect Neurosci. 2020 Mar 28;14(12):1273–83. doi: 10.1093/scan/nsaa001 (PMC7137720; doi:10.1093/scan/nsaa001)
Supplement: scan-18-207-File008_nsaa001 [file scan-18-207-file008_nsaa001.docx]

**Monitoring in Emotion Regulation: Behavioral Decisions and Neural Consequences**

**Shirel Dorman Ilan, Roni Shafir, Jeffrey L. Birk, George A. Bonanno & Gal Sheppes**

**Supplementary Materials**

**Self-reported affective consequences of monitoring regulatory decisions**

Prior to reporting additional analyses on the short-term self-reported affective consequences of monitoring regulatory choices, we note a combination of three design elements that make these results strongly underpowered. First, the post-choice self-report data analysis includes a total of 8 conditions (three factors, each with two levels): Emotional-Intensity (low, high), Initial-Implementation (Reappraisal, Distraction), and Monitoring-Choice (Switch, Maintain). Second, because self-reports are obtained following regulatory monitoring choices, it is not possible to control obtaining trials across all 8 conditions (a pre-condition to be included in the three-way interaction analysis). Third, based on this a-priori design decision, we collected post-choice implementation self-report scores on a very small fraction of trials (10%, i.e, a total of 18 trials). Considering these three elements of our design together, when we analyzed the data, we discovered that only 5 participants had rating values across all conditions. For all of these five participants, ratings in some conditions were based on a single trial.

Somewhat not surprisingly, when we analyzed the partial data of these five participants, we found no sign for an Emotional-Intensity x Initial-Implementation x Monitoring-Choice interaction on self-reports [*F*(1, 4)< 1, *p* = .80, η_p_^2^ =.02]. We believe the most plausible explanation for not obtaining the three-way interaction is that the experiment was not designed and thus strongly underpowered to detect findings associated with post-choice ratings.

***Replicating prior neural findings during initial implementation***

|  | Degree of Freedom | F | p | η_p_^2^ |
| --- | --- | --- | --- | --- |
| Emotional Intensity | 1,27 | 77.54 | .001** | .74 |
| Initial Implementation | 1,27 | 6.66 | .01* | .20 |
| Emotional Intensity X Initial Implementation | 1,27 | 3.36 | .07 | .11 |

Note: *<.05, **<.01

**Table S1.** Main and interaction effects of a 2 x 2 ANOVA with emotional intensity (high, low) and initial implementation (distraction, reappraisal) as repeated-measures factors, and the LPP as a dependent variable.

***First research question: Regulatory preferences predict regulatory choices to switch versus maintain an implemented strategy during post-implementation monitoring***

|  | Degree of Freedom | F | p | η_p_^2^ |
| --- | --- | --- | --- | --- |
| Emotional Intensity | 1,27 | 7.56 | .01** | 0.22 |
| Initial Implementation | 1,27 | 0.02 | .86 | 0.00 |
| Emotional Intensity X Initial Implementation | 1,27 | 60.99 | .001** | 0.69 |

Note: *<.05, **<.01

**Table S2.** Main and interaction effects of a 2 x 2 ANOVA with emotional intensity (high, low) and initial regulatory strategy (distraction, reappraisal) as repeated-measures factors, and switching frequency as a dependent variable.

***Second research question: Neuro-affective consequences (LPP modulation) of monitoring regulatory choices Linear Mixed Model (LMM) analysis***


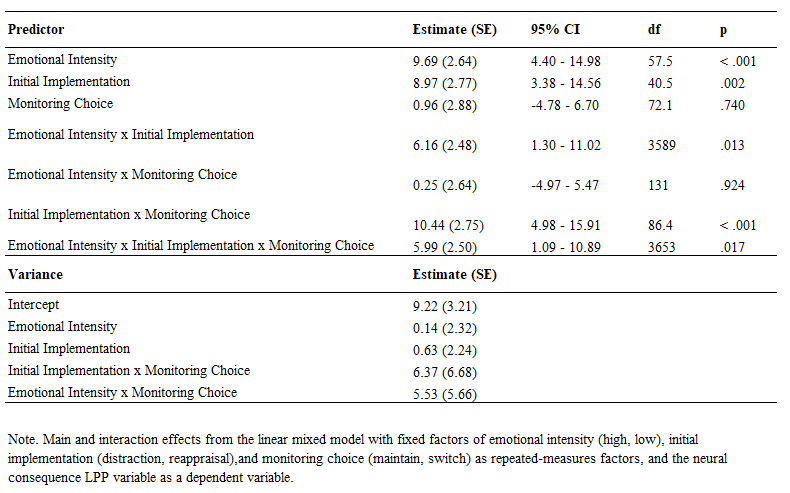


**Table S3.** Main and interaction effects of the final Linear Mixed Model (LMM) reported in the manuscript. Model consists all fixed effects together with the following random effect structure: intercept, Emotional-Intensity, Initial-Implementation, Initial-Implementation x Monitoring-Choice, and Emotional-Intensity x Monitoring-Choice.

**Two complimentary analyses of the second research question regarding Neuro-affective consequences (LPP modulation) of monitoring regulatory choices**

1. **An alternative “bottom up” Linear Mixed Models (LMM) analysis:**

In addition to the “top down” modelling approach described in the manuscript (i.e., starting with a *maximal* random effect structure and reducing complexity in subsequent modelling steps), a second “bottom-up” approach involves balancing parsimony with accuracy, by starting with the most *minimal* random effect structure (participant intercept only), followed by testing whether fit improves in models that increase in complexity in separate steps (i.e., whether model fit improves when adding random main effects, then random two-way interactions, and then random three-way interaction. c.f., Bell, Ene, Smiley, & Schoeneberger, 2013). Adopting this approach resulted in a final model that involved modeling only the participant intercept as a random effect. Specifically, the minimal random effect structure (i.e., modeling random intercept in addition to fixed effects) we began with, converged and yielded these model fit values (*-2*Log Likelihood (LL)* = 42,710.0). However, relative to this minimal random effect structure, none of the subsequent steps (random main effects; random two way interactions; random three way interaction) significantly improved model fit according to log-likelihood deviance comparison tests of each pair of models (all *Δ-2*LL* ≤ 2.4, all *df*s = 1, all *p*s ≥ .121).

Fully replicating the LMM analysis reported in the manuscript, we observed a significant three-way interaction of Intensity x Initial Strategy x Choice, *t*(4890) = -2.42; *B* = -5.95 µV; 95% CI = -10.78, -1.12; *p* = .016. Fully replicating our original follow-up tests, we showed that only in the high-intensity condition, there was a significant interaction of Initial Strategy x Choice, *t*(2396) = -4.56; *B* = -7.88 µV, 95% CI = -11.28, -4.49; *p* < .001. In contrast but consistent with our predictions and original findings, the second follow-up model showed that, in the low-intensity condition, there was no significant interaction of Initial Strategy x Choice, *t*(2413) = -1.15; *B* = -2.10 µV, 95% CI = -5.69, 1.48; *p* = .250. Decomposing the significant interaction under *high* intensity between initial strategy and choice confirmed predictions in showing that switching to reappraisal (*M* = -0.56, *SE* = 1.28) was associated with decreased LPP modulation, *t*(1099) = -2.64; *B* = -3.48 µV, 95% CI = -6.08, -0.89; *p* = .009, relative to maintaining distraction (*M* = 2.93, *SE* = 0.85). In addition, under high intensity, switching to distraction (*M* = 6.94, *SE* = 1.08) was associated with a greater LPP modulation, *t*(1135) = 3.62; *B* = 4.14 µV, 95% CI = 1.90, 6.38; *p* < .001, relative to maintaining reappraisal (*M* = 2.80, *SE* = 1.02).

**Conventional Analysis of Variance (ANOVA)**

To complement the “top down” LMM model reported in the manuscript and the “bottom up” LMM model reported above, we report here a conventional repeated-measures Analysis of Variance (ANOVA). Specifically, we conducted a 2 x 2 x 2 ANOVA with emotional intensity (high, low), initial implementation strategy (distraction, reappraisal), and monitoring regulatory choice (maintain, switch) as repeated-measures factors, and post-implementation LPP as the dependent variable.

Fully replicating the LMM analysis reported in the manuscript, and the “bottom up” LMM above, we found a significant Emotional Intensity x Initial Implementation x Monitoring Regulatory Choice interaction [*F*(1, 26) = 8.71, *p* = .006, η_p_^2^ =.25, Figure 4]. Decomposing the three-way interaction revealed results consistent with our prediction. That is, in *high* emotional intensity [*F*(1, 26) = 13.30, *p* = .001, η_p_^2^ = .34], but not in *low* emotional intensity [*F*(1, 26) = 1.26, *p* = .27, η_p_^2^ = .05], choosing to maintain or to switch to distraction, relative to maintain or switch to reappraisal, resulted in greater LPP modulation. Specifically, in high intensity, maintaining distraction following initial distraction implementation resulted in greater LPP modulation(*M* = 3.38, *SE* = 0.94), relative to switching to reappraisal (*M* = -1.54, *SE* = 1.83) [*F*(1, 26) = 6.18, *p* = .02, η_p_^2^ = 0.19]. Similarly, switching to distraction following initial reappraisal implementation resulted in greater LPP modulation (*M* = 7.48, *SE* = 1.17), relative to maintaining reappraisal (*M* = 3.55, *SE* = 1.34) [*F*(1, 26) = 8.86, *p* < .01, η_p_^2^ = 0.25]. Below we include additional information regarding all main and interaction effects (Table S4).

|  | Degree of Freedom | F | p | η_p_^2^ |
| --- | --- | --- | --- | --- |
| Emotional Intensity | 1,26 | 13.80 | .001** | .35 |
| Initial Implementation | 1,26 | 12.85 | .001** | .33 |
| Monitoring Choice | 1,26 | .09 | .768 | .00 |
| Emotional Intensity X Initial Implementation | 1,26 | 5.58 | .026* | .18 |
| Emotional Intensity X Monitoring Choice | 1,26 | .14 | .713 | .00 |
| Initial Implementation X Monitoring Choice | 1,26 | 10.41 | .003** | .28 |
| Emotional Intensity X Initial Implementation X Monitoring Choice | 1,26 | 8.71 | .006** | .25 |

Note: *<.05, **<.01

**Table S4.** Main and interaction effects following a 2 x 2 x 2 ANOVA with emotional intensity (high, low), initial regulatory strategy (distraction, reappraisal), and monitoring regulatory choice (maintain, switch) as repeated-measures factors, and the "neural consequence” LPP variable as a dependent variable.
